# Low birth weight and environmental tobacco smoke increases the risk of wheezing in adolescents: a retrospective cohort study

**DOI:** 10.1186/1471-2458-14-688

**Published:** 2014-07-05

**Authors:** Meng-Hung Lin, James L Caffrey, Yu-Sheng Lin, Pau-Chung Chen, Ching-Chun Lin, Wen-Chao Ho, Trong-Neng Wu, Ruey-Shiung Lin

**Affiliations:** 1 Department of Public Health, College of Public Health, China Medical University, Taichung, Taiwan; 2 Department of Integrative Physiology and Cardiovascular Research Institute, University of North Texas Health Science Center, Fort Worth, Texas, USA; 3 Department of Environmental and Occupational Health, University of North Texas Health Science Center, Fort Worth, Texas, USA; 4 Institute of Occupational Medicine and Industrial Hygiene, National Taiwan University College of Public Health, Taipei, Taiwan; 5 Institute of Epidemiology and Preventive Medicine, National Taiwan University College of Public Health, Taipei City, Taiwan; 6 Currently affiliated with National Center for Environmental Assessment, Office of Research and Development, U.S. Environmental Protection Agency, Washington, DC, USA

## Abstract

**Background:**

Low birth weight (LBW) and environmental tobacco smoke (ETS) exposure are each associated with wheezing in children. This study was designed to examine the combined association of LBW and ETS with wheezing.

**Methods:**

A retrospective birth cohort analysis linked with a national survey of allergic disorders among 1,018,031 junior high school students in Taiwan (1995–1996) was analyzed. The reported incidence of wheezing (yes or no) and ETS exposure (4 categories: 0, 1–20, 21–40 and greater than or equal to 41 household cigarettes per day) were obtained from validated questionnaires. Multiple logistic regression models were used to assess the associations of interest.

**Results:**

There were 844,003 (83%) subjects analyzed after the exclusion criteria. LBW was associated with an increased risk of reporting ever wheezing (odds ratio [OR] = 1.08, 95% confidence interval [CI] = 1.01–1.16), current wheezing (OR = 1.09, 95% CI = 1.00–1.20) and wheezing with exercise (OR = 1.11, 95% CI = 1.02–1.21) within the smoke-free cohort. Higher ETS exposure correlated to a higher risk of wheezing (ever, current and with exercise). With ETS exposure, adolescents from the lowest birth weight cohorts were more likely to report wheezing (ever, current and with exercise).

**Conclusions:**

ETS and LBW each has been related to increasing public health risk for respiratory symptoms among adolescents. Furthermore, LBW may aggravate the risk among those exposed to ETS. LBW, ETS and associated respiratory impairments may deserve special attention as part of a comprehensive environmental health risk assessment directed toward prevention and intervention.

## Background

A number of studies have linked low birth-weight to subsequent asthma in young children [1] and adolescents [2]. An association between environmental tobacco smoke (ETS) and asthma symptoms in children was initially proposed in 1993 [3]. It has since been documented that because ETS reduction probably led to decreased asthma hospitalization rate in children [4]. In addition to asthma, ETS can influence the likelihood of a variety of respiratory and allergic symptoms including wheezing, bronchitis, hay fever and eczema [5-7].

Most of the effects of premature birth on children have been documented, and long-term follow-up studies on respiratory symptoms in children born at term are rare. In the Prevention and Incidence of Asthma and Mite Allergy (PIAMA) birth cohort study [8], 3,628 children with a gestational age of 37 weeks or more were monitored for 7 years. Parental questionnaires were used to assess respiratory health yearly. The associations between birth weight, respiratory symptoms (wheezing, coughing, respiratory infections) and physician-diagnosed asthma were assessed in a repeated-event analysis. LBW was associated with a transient risk of respiratory symptoms before the age of 7 years. The effect on respiratory symptoms was again enhanced by ETS exposure. Collectively these observations led to the hypothesis that LBW and ETS exposure may have joint effects on wheezing risk in children. Thus, this study was designed to evaluate the relationships among LBW, ETS and wheezing in adolescents.

## Methods

### Study population

The study was based on a retrospective cohort, which was developed by linking the results of a nationwide respiratory health survey of junior high school children to the subjects’ respective birth records obtained from the Taiwan Birth Registry. The Taiwan Birth Registry was established in 1978 to collect key birth demographics including birth date, sex, parity, gestational age, birth weight and limited parental/environmental characteristics [9]. Respiratory screening was conducted by the National Taiwan University (NTU) and the Environmental Protection Administration in Taiwan (TEPA) over 6 months between October 1995 and March 1996.

**Figure 1:**
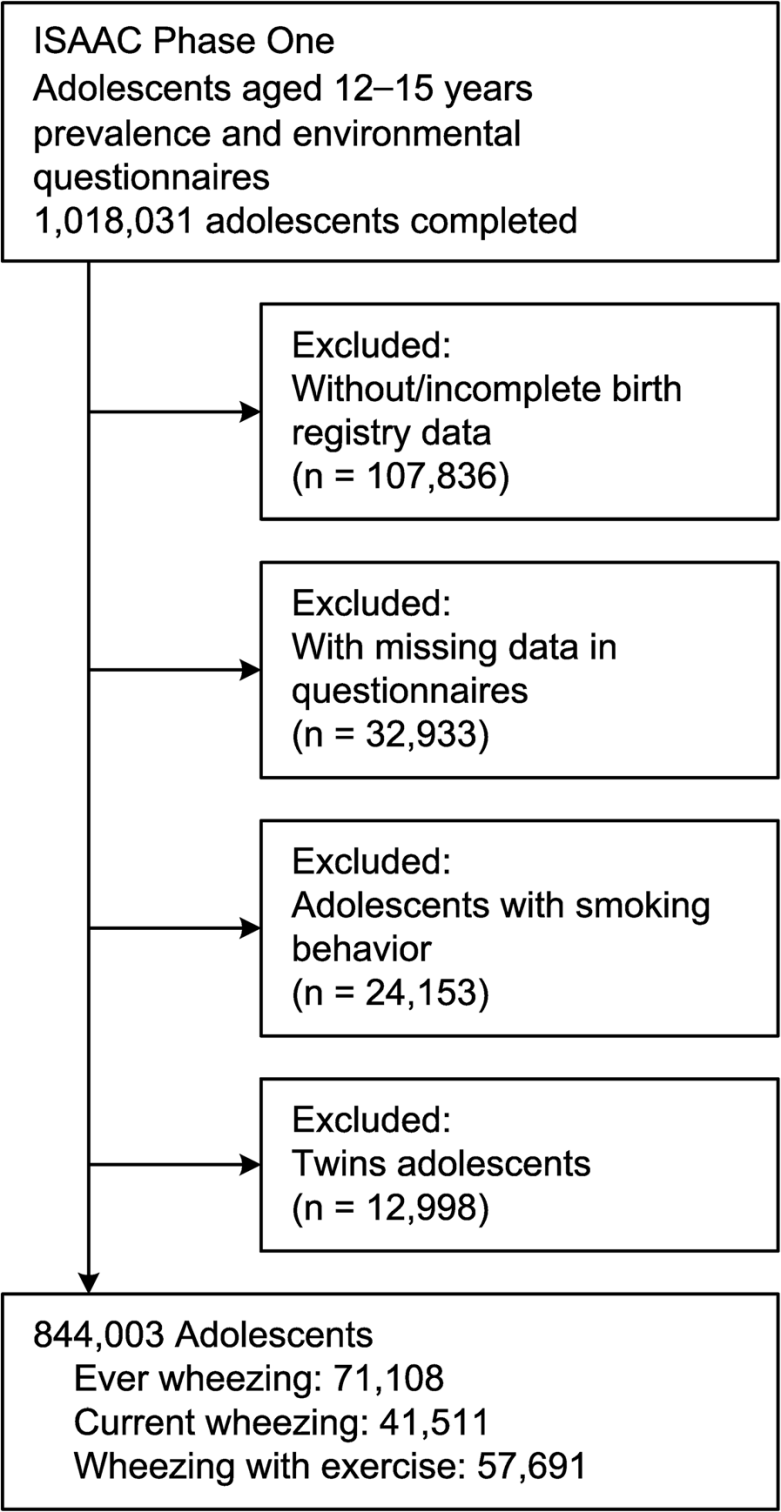
Schematic illustration of the study design.

Of the 1,139,452 junior high school students nationwide at the time, 1,018,031 (89.3%) students aged 12–15 years submitted completed International Study of Asthma and Allergies in Childhood (ISAAC) designed questionnaires, which were subsequently validated by computerized quality control programs [10]. This study has been reviewed and approved by the TEPA, the Institutional Review Board at NTU with written standard procedure and protocol for verbal consent of children and Ethics Committee approval. Because Institutional Review Board written consent was not required in Taiwan during 1995–1996, we also had verbal consent from children’ parents to agree to join this research and complete the questionnaires. Personal information was removed and remained anonymous during the entire study process. Records were excluded from the analysis for the following reasons: (1) incomplete birth registry data; (2) missing key questionnaire data; (3) participants currently smoking; and (4) twins (Figure 1).

### Definition of exposure

Information was collected regarding the current and past household smoking status of each participant’s adult household members and regular household visitors. The ETS exposure assessment was based on the question “How many cigarettes are smoked inside the house per day?” The answers included 4 categories: no smoking, 1–20, 21–40, and ≥41 cigarettes per household per day. To avoid the confounding effect of active smoking, 24,153 participants who were currently smoking were excluded.

### Definition of health outcomes

Ever wheezing, current wheezing and wheezing with exercise were identified by a positive answer to the following questions: “Has your child ever had wheezing or whistling in the chest, and shortness of breath at any time in the past?”; “Has your child had wheezing or whistling in the chest, and shortness of breath during the past 12 months?” and “Has your child’s chest sounded wheezy during or after exercise during the past 12 months?” [11,12]. Physician-diagnosed respiratory impairments were defined by the parents’ reports of whether their child had ever been diagnosed with asthma and/or rhinitis by a physician.

### Birth weight and gestational age measurement

The birth condition is based on Taiwan Birth Registry. Three birth weight groups were categorized (<2500 g, 2500–4000 g, and ≥4000 g). Two gestation age groups were also categorized (<37 weeks and ≥37 weeks). Furthermore, for birth order information, 4 groups were divided (1, 2, 3 and ≥4).

### Statistical analysis

Adjusted odds ratios (ORs) and 95% confidence interval (CI) were estimated by multiple logistic regression models for wheezing (yes or no) associated with birth weight, ETS and potential risk factors (sex, age, parental education, gestation age and birth order). Further joint effects of birth weight and ETS regarding adolescent wheezing were assessed after adjusting for potential risk factors. A sensitivity analysis was conducted for adolescents without physician-diagnosed asthma or rhinitis. SAS software, version 9.3, was used in the analysis (SAS Institute, Inc., Cary, NC). All of the reported *P*-values were based on two-tailed assumptions. Statistical significance was set at 0.05.

## Results

There were 844,003 surveys available for the current analyses after the exclusion criteria were applied (Figure 1). The rates of adolescents who had ever wheezed, who currently wheezed and who wheezed with exercise were 7.0%, 4.1% and 6.5% for female subjects and 9.6%, 5.8% and 7.1% for male subjects, respectively. Higher ETS exposure had higher rate of wheezing with exercise. In the meantime, the two highest ETS exposure groups were associated with having the top two ever and current wheezing rates. Wheezing was more frequently observed in younger students, and those from the LBW (<2500 g), and preterm (<37 weeks) cohorts. Higher parental education was associated with a greater rate of ever and current wheezing in their children but a lower rate of wheezing with exercise. Higher birth order (younger siblings) similarly had a lower rate of ever and current wheezing, but not wheezing with exercise (Table 1).

**Table 1:** Demographic characteristics of the subjects with wheezing

**Variables**	**Total no.**	**Proportion of wheezing, %**		
		**Ever wheezing**	**Current wheezing**	**Wheezing with exercise**
Sex				
Female	428529	7.0	4.1	6.5
Male	415474	9.6	5.8	7.1
Age (years)				
12	193263	8.8	5.4	7.4
13	287802	8.8	5.2	7.0
14	285288	8.0	4.5	6.5
15	77650	7.5	4.3	6.2
Parental education				
Elementary or less	185241	6.4	4.1	7.2
Junior high	221062	9.0	4.4	7.1
Senior high	289858	8.7	5.1	6.6
College or above	147842	12.7	6.4	6.4
Household cigarettes (per day)				
0	367810	8.8	4.9	6.2
1–20	424149	7.9	4.8	7.0
21–40	40090	9.3	5.9	9.0
≥41	11954	10.1	7.1	11.3
Birth weight (g)				
<2500	25337	9.3	5.4	7.8
2500–3999	764963	8.4	4.9	6.8
≥4000	53703	8.0	4.7	7.0
Gestation age (weeks)				
<37	17752	10.1	5.8	7.4
≥37	826251	8.4	4.9	6.8
Birth order				
1	326622	9.7	5.6	6.8
2	270237	8.5	4.8	6.7
3	161474	7.1	4.3	6.9
≥4	85670	6.2	3.9	7.2
Ever physician-diagnosed asthma				
Yes	39528	77.0	47.3	37.7
No	804415	5.1	2.8	5.3
Ever physician-diagnosed rhinitis				
Yes	185811	20.3	12.3	11.9
No	658192	5.1	2.8	5.4
Ever physician-diagnosed asthma or rhinitis				
Yes	200800	23.9	14.4	13.5
No	643203	3.6	2.0	4.7

As compared with normal birth weight (2500–4000 g), LBW was associated with an increased risk of having ever wheezed (OR = 1.07, 95% CI = 1.02–1.12), current wheezing (OR = 1.07, 95% CI = 1.003–1.14) and wheezing with exercise (OR = 1.16, 95% CI = 1.10–1.22). ETS exposure was progressively associated with higher adjusted ORs for symptoms of wheezing. For the highest ETS exposure group, the increased risk was as follows: having ever wheezed (OR = 1.36, 95% CI = 1.28–1.44), current wheezing (OR = 1.66, 95% CI = 1.54–1.78) and wheezing with exercise (OR = 1.90, 95% CI = 1.80–2.02) (Table 2).

**Table 2:** Adjusted odds ratios (OR) and 95% confidence intervals (95% CI) for wheezing in Taiwanese adolescents

**Variables**	**Adjusted odds ratio (95% CI)**		
	**Ever wheezing**	**Current wheezing**	**Wheezing with exercise**
Sex			
Female	1.00 (Reference)	1.00 (Reference)	1.00 (Reference)
Male	1.46 (1.44–1.49)	1.44 (1.41–1.47)	1.09 (1.08–1.11)
Age (years)			
12	1.13 (1.09–1.16)	1.25 (1.20–1.30)	1.22 (1.18–1.26)
13	1.13 (1.10–1.17)	1.21 (1.17–1.26)	1.15 (1.11–1.18)
14	1.04 (1.01–1.08)	1.04 (1.00–1.08)	1.05 (1.02–1.09)
15	1.00 (Reference)	1.00 (Reference)	1.00 (Reference)
Parental education			
Elementary or less	1.00 (Reference)	1.00 (Reference)	1.00 (Reference)
Junior high	1.04 (1.01–1.06)	1.01 (0.97–1.04)	0.97 (0.95–1.00)
Senior high	1.28 (1.25–1.31)	1.17 (1.13–1.20)	0.92 (0.89–0.94)
College or above	1.93 (1.88–1.98)	1.49 (1.44–1.54)	0.91 (0.89–0.94)
Household cigarettes (per day)			
0	1.00 (Reference)	1.00 (Reference)	1.00 (Reference)
1–20	0.98 (0.96–1.00)	1.05 (1.02–1.07)	1.13 (1.11–1.15)
21–40	1.21 (1.16–1.25)	1.35 (1.29–1.41)	1.48 (1.43–1.54)
≥41	1.36 (1.28–1.44)	1.66 (1.54–1.78)	1.90 (1.80–2.02)
Birth weight (g)			
<2500	1.07 (1.02–1.12)	1.07 (1.003–1.14)	1.16 (1.10–1.22)
2500–3999	1.00 (Reference)	1.00 (Reference)	1.00 (Reference)
≥4000	0.96 (0.93–0.99)	0.96 (0.92–1.00)	1.02 (0.99–1.06)
Gestation age (weeks)			
<37	1.14 (1.08–1.21)	1.12 (1.04–1.20)	1.02 (0.95–1.09)
≥37	1.00 (Reference)	1.00 (Reference)	1.00 (Reference)
Birth order			
1	1.00 (Reference)	1.00 (Reference)	1.00 (Reference)
2	0.89 (0.88–0.91)	0.88 (0.86–0.90)	0.99 (0.97–1.01)
3	0.79 (0.78–0.81)	0.81 (0.79–0.84)	1.00 (0.97–1.02)
≥4	0.74 (0.72–0.77)	0.77 (0.74–0.80)	1.03 (1.00–1.06)

As evidence of an independent effect, LBW specifically generated higher ORs within the smoke-free cohort for ever wheezing (OR = 1.08, 95% CI = 1.01–1.16), current wheezing (OR = 1.09, 95% CI = 1.00–1.20) and wheezing with exercise (OR = 1.11, 95% CI = 1.02–1.21). Furthermore, those in the LBW cohort had the highest risk of ETS exposure related ever wheezing (OR = 1.73, 95% CI = 1.28–2.33), current wheezing (OR = 2.20, 95% CI = 1.56–3.09) and wheezing with exercise (OR = 2.86, 95% CI = 2.18–3.75) (Table 3). A joint effect was observed between LBW and the highest ETS exposure group regarding wheezing.

**Table 3:** Association between household cigarettes, birth weight, and wheezing status in adolescents

Adjusted OR^*^	**Household cigarettes (per day)**			
**(95% CI)**	**0**	**1–20**	**21–40**	**≥41**
**Ever Wheezing**				
Birth weight (g)				
<2500	1.08 (1.01–1.16)	1.03 (0.97–1.10)	1.24 (1.03–1.49)	1.73 (1.28–2.33)
2500–3999	1.00 (Reference)	0.98 (0.97–1.00)	1.21 (1.17–1.26)	1.36 (1.27–1.45)
≥4000	0.98 (0.93–1.03)	0.92 (0.88–0.97)	1.17 (1.02–1.34)	1.17 (0.91–1.50)
**Subgroup: excluded ever physician-diagnosed asthma cases**				
Birth weight (g)				
<2500	1.06 (0.97–1.17)	1.12 (1.03–1.22)	1.42 (1.14–1.78)	2.11 (1.49–2.98)
2500–3999	1.00 (Reference)	1.05 (1.03–1.08)	1.38 (1.31–1.44)	1.60 (1.48–1.73)
≥4000	0.98 (0.92–1.05)	0.99 (0.94–1.06)	1.29 (1.09–1.52)	1.43 (1.07–1.90)
**Subgroup: excluded ever physician-diagnosed rhinitis cases**				
Birth weight (g)				
<2500	1.05 (0.95–1.17)	1.10 (1.00–1.20)	1.48 (1.17–1.88)	2.12 (1.44–3.12)
2500–3999	1.00 (Reference)	1.07 (1.04–1.09)	1.43 (1.36–1.51)	1.56 (1.43–1.70)
≥4000	0.97 (0.91–1.05)	1.01 (0.94–1.07)	1.24 (1.03–1.49)	1.24 (0.90–1.72)
**Subgroup: excluded ever physician-diagnosed asthma or rhinitis cases**				
Birth weight (g)				
<2500	1.02 (0.89–1.16)	1.15 (1.03–1.28)	1.56 (1.18–2.06)	2.20 (1.41–3.44)
2500–3999	1.00 (Reference)	1.10 (1.07–1.14)	1.54 (1.45–1.63)	1.72 (1.57–1.90)
≥4000	1.00 (0.92–1.09)	1.07 (0.99–1.15)	1.27 (1.03–1.58)	1.42 (0.99–2.03)
**Current Wheezing**				
Birth weight (g)				
<2500	1.09 (1.00–1.20)	1.08 (1.00–1.18)	1.47 (1.18–1.84)	2.20 (1.56–3.09)
2500–3999	1.00 (Reference)	1.05 (1.03–1.07)	1.33 (1.27–1.40)	1.66 (1.37–1.79)
≥4000	0.98 (0.92–1.04)	0.99 (0.93–1.05)	1.47 (1.25–1.72)	1.45 (1.09–1.93)
**Subgroup: excluded ever physician-diagnosed asthma cases**				
Birth weight (g)				
<2500	1.07 (0.94–1.22)	1.16 (1.04–1.30)	1.94 (1.49–2.52)	2.57 (1.69–3.89)
2500–3999	1.00 (Reference)	1.14 (1.10–1.17)	1.52 (1.43–1.61)	1.97 (1.80–2.16)
≥4000	1.00 (0.92–1.09)	1.08 (1.00–1.16)	1.62 (1.33–1.97)	2.00 (1.45–2.77)
**Subgroup: excluded ever physician-diagnosed rhinitis cases**				
Birth weight (g)				
<2500	1.06 (0.92–1.22)	1.16 (1.03–1.30)	1.82 (1.36–2.43)	2.91 (1.88–4.50)
2500–3999	1.00 (Reference)	1.15 (1.11–1.19)	1.62 (1.52–1.72)	1.99 (1.80–2.20)
≥4000	1.01 (0.92–1.11)	1.06 (0.97–1.15)	1.51 (1.21–1.89)	1.50 (1.02–2.23)
**Subgroup: excluded ever physician-diagnosed asthma or rhinitis cases**				
Birth weight (g)				
<2500	0.98 (0.81–1.18)	1.24 (1.07–1.43)	2.19 (1.57–3.04)	2.79 (1.63–4.78)
2500–3999	1.00 (Reference)	1.21 (1.16–1.26)	1.74 (1.61–1.88)	2.22 (1.98–2.49)
≥4000	1.07 (0.95–1.20)	1.14 (1.03–1.26)	1.60 (1.23–2.07)	1.70 (1.09–2.66)
**Wheezing with Exercise**				
Birth weight (g)				
<2500	1.11 (1.02–1.21)	1.33 (1.24–1.43)	1.66 (1.38–2.00)	2.86 (2.18–3.75)
2500–3999	1.00 (Reference)	1.13 (1.11–1.16)	1.49 (1.43–1.55)	1.89 (1.78–2.01)
≥4000	1.06 (1.01–1.12)	1.13 (1.08–1.19)	1.46 (1.28–1.67)	1.81 (1.45–2.26)
**Subgroup: excluded ever physician-diagnosed asthma cases**				
Birth weight (g)				
<2500	1.17 (1.01–1.23)	1.38 (1.28–1.49)	1.79 (1.45–2.20)	3.35 (2.51–4.47)
2500–3999	1.00 (Reference)	1.18 (1.16–1.21)	1.58 (1.51–1.65)	2.06 (1.93–2.21)
≥4000	1.08 (1.02–1.15)	1.21 (1.14–1.27)	1.48 (1.27–1.73)	2.01 (1.58–2.56)
**Subgroup: excluded ever physician-diagnosed rhinitis cases**				
Birth weight (g)				
<2500	1.11 (0.99–1.23)	1.32 (1.21–1.43)	1.84 (1.48–2.30)	2.74 (1.94–3.87)
2500–3999	1.00 (Reference)	1.18 (1.15–1.21)	1.59 (1.52–1.67)	2.02 (1.87–2.17)
≥4000	1.07 (0.99–1.14)	1.20 (1.13–1.27)	1.41 (1.19–1.67)	1.92 (1.48–2.50)
**Subgroup: excluded ever physician-diagnosed asthma or rhinitis cases**				
Birth weight (g)				
<2500	1.10 (0.98–1.24)	1.33 (1.21–1.43)	1.90 (1.50–2.40)	2.87 (1.99–4.13)
2500–3999	1.00 (Reference)	1.20 (1.17–1.21)	1.60 (1.53–1.69)	2.10 (1.94–2.27)
≥4000	1.09 (1.02–1.18)	1.23 (1.15–1.27)	1.42 (1.18–1.70)	1.97 (1.49–2.60)

^*^Adjusted for sex, age, parental education, gestation age, birth order.

A sensitivity analysis was conducted in Table 3 to assess and exclude the potential effects of changes in ETS exposure patterns following physician diagnoses of asthma and/or rhinitis. After excluding adolescents with clinical diagnoses, consistent dose–response curves for ETS exposure and the risk of ever wheezing were found for all three birth weight groups (low birth weight, normal birth weight and high birth weight). Furthermore, low birth weight still had the highest risk of ETS exposure related wheezing at any time. The potential joint effect between low birth weight and the highest ETS exposure group in those who had ever wheezed was similar to that described narrowing the cohort.

A joint effect between LBW and the highest ETS exposure group in those who currently wheezed was also found. Dose–response relationships for ETS exposure and the risk of current wheezing were found for low birth weight and normal birth weight after excluding adolescents who were diagnosed with asthma and/or rhinitis. The high birth weight group retained a dose–response relationship after excluding those diagnosed with asthma and/or rhinitis. Again, low birth weight had the highest risk of current ETS exposure related wheezing. The joint effect between low birth weight and ETS exposure appeared to emerge at the second highest ETS exposure category.

Dose-responses of wheezing with exercise and ETS were found before and after excluding physician diagnosed asthma and/or rhinitis. A joint effect was observed in two higher ETS exposure categories but suggestions of an effect lower ETS exposure were evident.

## Discussion

This study shows that LBW and ETS have the independent and joint effects on increasing adolescents wheezing rate. The low birth weight cohorts were more likely to report wheezing among adolescents [1,2,13,14]. Regardless of birth weight, higher ETS exposure consistently resulted in increased odds of wheezing, as reported by others [3,4]. LBW and ETS exposure each pose a genuine respiratory risk. ETS is clearly a serious factor. However, regardless of ETS exposure, the risk for symptoms in the LBW cohort was greater than for their normal birth weight and heavy birth weight counterparts. Thus, ETS exposure appears to exaggerate the negative influence of LBW specifically on wheezing.

The mechanism explaining the interplay between ETS and LBW on wheezing is both curious and unclear. Seymour et al. reported that exposure of ovalbumin-sensitized mice to ETS elicited exaggerated IgE, IgG1, eosinophils and Th2 cytokines (particularly IL-4; IL-10) responses. The combined sensitization to allergens and added effect of ETS on Th2 responses may have been responsible for the prevalence of allergic symptoms in individuals with tobacco smoke exposure. Tobacco smoke can worsen atopic conditions by irritating the skin and mucous membranes, thus facilitating the access of allergens and the opportunity for sensitization [15].

Furthermore, oxidative stress associated with the exposure to cigarette smoke could act epigenetically via pro-inflammatory genes by altering transcription factors (e.g. nuclear factor κB, NF-κB) and histones or by remodeling chromatin. In children born at LBW, cigarette smoke could contribute to reduced histone deacetylase activity. Reductions in this key transcriptional moderator can favor the activation of NF-κB and the expression of the proinflammatory cytokines IL-6 and IL-8 in susceptible immature lung tissue, which may later lead to the development of asthma [16].

Whether the increased vulnerability associated with LBW is the result of subtle changes in prenatal programming or the reactive adjustments of less immunologically mature infants to the post-natal environment is unclear. Although understanding the cause will obviously facilitate the finding of an appropriate remedy, it is very clear that children with LBW should be carefully monitored for early signs of wheezing and allergic disorders. Despite these caveats, it is abundantly clear that exposure to ETS is a significant threat to the health of all children.

The strength of the current study is derived from its large, representative national sample, which should provide an unbiased estimation of risk. The study is the first to address the joint effects between birth weight, ETS and wheezing symptoms (ever, current and with exercise). Increasing ETS exposure was a factor in the expression of wheezing symptoms, especially in subjects with LBW. Consistent dose–response effects were observed.

Maternal smoking but not paternal smoking during pregnancy was related to LBW and preterm delivery [17]. Maternal smoking during pregnancy was also related to wheezing [18]. The ETS exposure estimates in Taiwan are high (≥49.0%) among children but surprisingly lower in maternal smoking during pregnancy (≤3.9%) [17,18]. However, the ETS dose–response effect was quite robust, suggesting that there is no safe exposure. These data should lend additional support to on-going public health campaigns to limit childhood exposure to ETS.

There were several potential limitations in the analysis. Wheezing symptoms and ETS were estimated cross-sectionally, and no specific prenatal, during pregnancy or perinatal ETS exposure data were available. The cross-sectional study provided little information about the causal relationship between ETS and wheezing symptoms. Prenatal maternal smoking (or prenatal ETS exposure) has been proposed as a contributing factor to LBW [17,19-21]. LBW might, in fact, serve as an index outcome of prenatal maternal smoking. Prenatal ETS and LBW also showed the independent and joint effects on wheezing and asthma during childhood [19]. A pooled analysis showed that both prenatal and postnatal ETS were independently related to wheezing [22]. For LBW adolescents, ETS exposure could play an important and consistent role in increasing the risk of wheezing. Moreover, this study used questionnaires, and objective data on ETS exposure were unavailable because cotinine measurements were impractical in such a large population. However, self-reported smoke exposure correlates well with measured cotinine [23]. As a result, several studies have successfully applied self-reported exposure without measuring cotinine [5,18]. Finally, the source of the diagnoses (questionnaire vs. physician) might have biased the magnitude of the estimated increase in prevalence. However, the multiple variable regression model used to correlate ETS with allergic diseases was controlled for physician diagnosis; thus, the increases in risk appear to be a genuine concern.

## Conclusions

In summary, this study provides an unbiased estimate association within a large national sample. An increased risk of wheezing is related to LBW children. A significant dose–response association between ETS exposure and the rate of wheezing was clearly demonstrated. LBW and ETS exposure could have significant independent and joint effects on respiratory health in smoking-free adolescents. We recommend that adolescents should avoid ETS exposure to reduce the risk of wheezing, especially for those born with LBW.

## Abbreviations

LBW: Low birth weight; ETS: Environmental tobacco smoke; OR: Odds ratio; CI: Confidence interval.

## Competing interests

The authors declare that they have no competing interests.

## Authors’ contributions

WCH, PCC, TNW, RSL: conception and survey design. MHL, JCL, YSL, CCL: conducted the statistical analyses. MHL, WCH, JLC, YSL: interpretation of the data, drafting the manuscript. All authors contributed to the, revised the article critically for important intellectual content, and approved the final manuscript. All authors read and approved the final manuscript.

## Pre-publication history

The pre-publication history for this paper can be accessed here:

http://www.biomedcentral.com/1471-2458/14/688/prepub
